# Does Online Implementation Make a Difference in the Effects of a Mental Health Curriculum at Schools?

**DOI:** 10.3390/ijerph192416990

**Published:** 2022-12-17

**Authors:** Margarida Frade dos Santos, Celeste Simões, Anabela Caetano Santos, Paula Lebre, Ilaria Grazzani

**Affiliations:** 1 Faculdade de Motricidade Humana, Universidade de Lisboa, 1495-751 Lisbon, Portugal; 2 Instituto de Saúde Ambiental, Faculdade de Medicina, Universidade de Lisboa, 1649-028 Lisbon, Portugal; 3 Instituto de Etnomusicologia INET-MD, Faculdade de Motricidade Humana, Universidade de Lisboa, 1495-751 Lisbon, Portugal; 4 Department of Human Science for Education “R. Massa”, University of Milano-Bicocca, 20126 Milan, Italy

**Keywords:** digital mental health, well-being, social and emotional learning, COVID-19, PROMEHS, online, program, education

## Abstract

COVID-19 changed and challenged education, with schools obliged to adapt to online settings. This study aims to evaluate the impact of a mental health curriculum implemented at schools, considering the implementation settings: online, onsite, and mixed (online and onsite). From kindergarten to high school, 933 students were evaluated by teachers regarding their social and emotional learning, strengths and difficulties, and academic outcomesin two measuring times: pre- and post-test. A qualitative analysis of teachers’ adaptations to the online implementation was also conducted. Results revealed a positive impact with both mixed and onsite implementation. However, the mixed format demonstrated significant positive changes between the pre—and post-test, namely in relationship skills, responsible decision-making, internalized problems, and academic achievement. The mixed format with few online activities appears to have a more positive impact on students. Nevertheless, implementing social and emotional skills (SES) activities exclusively online seems to positively affect some SES domains more than onsite and mixed formats. Teachers used synchronous (e.g., digital platforms) and asynchronous (e.g., extra resources) adaptations for the implementation. This study shows that implementing mental health programs at schools, in this case, PROMEHS, is beneficial for students, even amidst the pandemic, and regardless of the implementation settings.

## 1. Introduction

With the COVID-19 pandemic, the world was challenged in many ways that implied multiple changes in all contexts, with consequences that affected the population’s mental health [1,2,3]. Specifically, in education systems, the pandemic originated challenges that had to be addressed without preparation or planning due to the lockdowns, including the total adaptation to an online learning approach [4,5,6]. Subjects, activities, and programs designed to be implemented onsite had to be adapted. Teachers struggled due to a lack of experience in online teaching, little preparation time, and proper skills [7,8].

Research about the COVID-19 impact on mental health has been widely active, with cross-sectional studies depicting the periods of the lockdown being the most published/present, lacking studies with longitudinal designs covering the periods before, during, and after lockdown periods [9]. Regarding children and adolescents, meta-analyses and systematic reviews have reported an increased manifestation of internalized (e.g., anxiety and depression) and externalized (e.g., aggressiveness) problems, relationship problems, sleep problems, reduction of life satisfaction, and wellbeing [1,10,11,12,13,14,15]. Not only has the pandemic threatened children and adolescents’ mental health, but also the development of their social and emotional skills [9,14,16]. In a study by Martinsone and colleagues [9], students from Latvia, Italy, and Portugal were evaluated in two time periods during COVID-19 and lockdown measures. Results revealed an increase in internalized and externalized problems and a reduction in social and emotional skills [9]. Their study also found a significant negative correlation between adolescents’ social and emotional skills and internalized and externalized problems. Therefore, social and emotional skills can act as a protective factor and resilience, reducing the impact of COVID-19 and the manifestation of internalized and externalized problems [14,17].

Before the pandemic, the World Health Organization reported that, worldwide, 1 in 8 children and adolescents between 6- and 18-years-old experienced a mental health problem and that fifty percent of these problems began at 14 [14]. Preventive measures and intervention programs focused on reducing the risk of future problems were developed, implemented, and evaluated to address various areas, such as mental health, wellbeing, social and emotional skills, and resilience, with schools considered the ideal context for the implementation [14,18,19]. The benefits associated with implementing these programs at school are [19,20,21,22,23]: an improvement in self-efficacy, wellbeing, academic achievement, and the relationship with oneself and others; protection of at-risk students; and decreased behavioral problems, conflicts, and emotional problems.

In pre-pandemic conditions, mental health programs implemented in the school context had a considerate positive impact with a recognition of the importance of integrating social and emotional skills into the school curriculum [19]. With COVID-19 lockdowns and its already identified implications on children and adolescents’ mental health, it is even more critical to implement these programs, for example, during lockdown periods with online school. The implementations can occur by developing programs and interventions or adapting existing ones to the online context, with further evaluation of their impact [2,24,25,26,27].

Programs and interventions implemented online, using digital technologies, platforms, or apps, have been used in and out of the school context to promote mental health [28,29,30,31,32,33]. However, most of these programs in the school context are: self-paced, students do it during breaks; are implemented by other professionals (e.g., psychologists, school counselors, health professionals) that are not the teacher; or the teacher intervention is only to present the program and to give support regarding the platform use, but they are not involved through the process [34,35,36,37]. The results of a systematic review indicated that online digital programs with high levels of human interaction were the most effective [37]. Taking this into account, the intervention by a teacher might be a determinant of students’ motivation and engagement during the program, also having a higher impact on the expected outcomes [38,39]. However, research on online learning entails mostly the subjects of the traditional school curriculums, disregarding program implementation at schools by teachers around the themes of mental health, resilience, and social and emotional skills [40,41].

To our knowledge, research around the online adaptations of social and emotional skills and mental health programs in the school context is scarce, especially for the pandemic period, with only a few studies being published. For example, Currie and colleagues [42] examined the online learning adaptations teachers applied when delivering the program Second Step. Although accessed via an online platform, this program was developed to be implemented onsite (i.e., in class) [42]. A toolbox was created for online adaptations in collaborative work between elementary school teachers and researchers [42]. Adaptations around the categories defined included: the use of breakout rooms; using the chat window to share with the whole class; using online discussion boards or other platforms; using paper to write or draw; using non-verbal signs or sticky notes; performing roleplays for the camera followed by group discussion; recording the scene and then show in class; and maintaining the original activity if students had room to move when activities included movement [42]. Additionally, instead of using handouts for each student, teachers asked them to answer verbally or use collaborative tools to share and submit answers, pictures, work, or other documents [42].

A social and emotional learning program entitled Adventures Aboard the S.S. GRIN was adapted to online school learning amidst COVID-19 and implemented in third-grade students [43]. The program revealed an increment of social and emotional skills after the implementation [43].

Chen and Adams [44] interviewed preschool teachers to identify strategies implemented in online schooling to promote children’s social and emotional development. The strategies more frequently mentioned were reading a book followed by a discussion, the use of visuals, and engagement in targeted conversations [44]. Teachers also noted that with remote learning, they expanded their teaching tools [44].

The support from schools and teachers was even more critical during the lockdown to help children and adolescents develop skills and strategies to cope with those stressful events [42]. That can be provided with programs that develop those skills [24], with digital technologies crucial for maintaining the interaction between peers and teachers and allowing the continuity of learning through online school [14]. Nevertheless, implementing these programs at the school’s reopening is crucial to support students and answer their social and emotional needs, to minimize the consequences of the lockdown and the overall pandemic [24,45].

The present study aims to contribute to evidence on the impact of mental health programs at schools while addressing gaps regarding the knowledge on the impact of a program implementation in different school learning settings since they are mostly implemented onsite, with little research about online implementation. Although programs’ adaptations to online settings have been published, little is known about the adaptations’ effects on the program’s impact. The study also addresses a literature gap on the program’s impact when implemented during COVID-19 and in the school context. It also answers directions by the scientific community to evaluate programs implemented in this period. Therefore, the present study aims to investigate the impact of a mental health curriculum at schools implemented by teachers during COVID-19, considering different implementation settings. More specifically, this study aims to contribute to the understanding of the impact of the implementation of the PROMEHS curriculum on mental health, social and emotional skills, and academic outcomes, considering the settings of implementation, namely online, onsite, and mixed (online and onsite), which occurred due to the restrictions imposed by the pandemic. It also aims to evaluate if the results of the mixed implementation differ considering the number of online activities implemented and the school levels. Lastly, we aim to identify the online digital adaptations teachers made to the curriculum developed for onsite implementation.

## 2. Materials and Methods

### 2.1. Study Design

This study is part of an Erasmus + Project, PROMEHS—Promoting Mental Health at Schools, that developed, implemented, and evaluated an evidence-based universal curriculum to promote students’ mental health from ages 3 to 18. PROMEHS was the first mental health curriculum developed in collaboration between researchers, policymakers, and scientific institutions from seven European countries (Italy, Croatia, Greece, Latvia, Malta, Portugal, and Romania). It was a quasi-experimental study with experimental and waiting groups evaluated in two measuring times (pre- and post-test). Between measuring times, the experimental group had teachers’ training and curriculum implementation at school and online. In contrast, the waiting group had the teachers’ training and implementation after the post-test.

Students were evaluated by three sources: teachers, parents, and themselves. Students’ self-report started at age 8. This study only includes data collected in Portugal, specifically the teachers’ evaluations of students from the experimental group.

At the beginning of the school year of 2020/2021, the research team scheduled meetings with policymakers and wellness organizations, who then contacted school principals who could be interested in PROMEHS. Then, each school principal contacted teachers to assess their interest in participating. After the selection of schools, meetings were scheduled with teachers and school psychologists to present the project and the evaluation process. Informed consents were sent to parents through the teachers. The collected consents were kept in the schools, and the research team created an identification code for each student that teachers further completed to guarantee confidentiality and anonymity. This code was used in both moments of evaluations and by the three sources of students’ evaluations. The links for the online surveys were sent via e-mail to teachers, who then shared them with the parents and students. Students completed the survey in class and gave their assent online before starting the survey. However, some had to be completed at home due to the lockdown imposed in January 2021. Due to limitations at schools or home, some surveys were collected using paper versions, which schools then sent to the research team, who inserted the data into the database. The pre-test was between December 2020 and mid-February 2021, and the post-test was between the end of May 2021 and mid-July 2021. Between evaluations, teachers undertook a total of 25 h of training (16 h were undertaken before the implementation and started in January 2021, and 9 h were supervision during the implementation) given by the research team. After the first part of the training, teachers had to implement in class a minimum of twelve activities of the curriculum (four of each theme that comprises PROMEHS: Promoting Social and Emotional Learning; Promoting Resilience; and Preventing Social, Emotional and Behavioral Problems). For more information about the PROMEHS project and methodology procedures, see Cefai and colleagues’ study [46].

The PROMEHS project was developed between February 2019 and August 2022. In Portugal, the implementation phase began in February of 2021, corresponding to a lockdown phase. Facing these situations, the research team allowed teachers two options: starting to implement during the lockdown and creating adaptations for the activities to enable online implementation or waiting until the lockdown was over and beginning the implementation onsite.

### 2.2. Participants

For the quantitative analysis, a total of 1134 students from kindergarten to high school were evaluated by teachers. A total of 201 were eliminated due to having more than fifty percent of missing values [47], namely students that were not assessed on pre- or post-test. The final sample included 933 students evaluated in both pre- and post-test. 

Data from a total of 55 teachers were used in the qualitative analysis. From these, 35 used a mixed method (online and onsite implementation), and 20 used an onsite method.

### 2.3. Measures and Procedures

Quantitative analysis:

Teachers completed the Strengths and Difficulties Questionnaire (SDQ) [48], the Social Skills Improvement System Social-Emotional Learning Brief Scales (SSIS-SEL) [49,50,51], and an Academic Engagement scale. 

*Mental Health*: The SDQ [48] assesses children and adolescents’ mental health by measuring behavioral and emotional difficulties. The SDQ comprises 25 items organized in five scales (five items each). Four scales are related to difficulties (emotional symptoms, behavioral problems, hyperactivity, and peer problems), and one to strengths (prosocial behavior). A 3-point Likert scale ranging from 0 (not true) to 2 (certainly true) is used. Higher scores on the difficulties scales mean more significant difficulties, and on the strengths scale, higher prosocial behavior. In this study, the model that considers internalized (i.e., emotional symptoms and peer problems scales), externalized problems (i.e., conduct problems and hyperactivity scales), and prosocial behavior was used [52]. Cronbach’s alpha reported acceptable to good internal consistency (internalized problems: α_pre_ = 0.78, α_post_ = 0.80; externalized problems: α_pre_ = 0.87, α_post_ = 0.87; prosocial behavior: α_pre_ = 0.87, α_post_ = 0.87). 

*Social and Emotional Skills* (SES): The SSIS-SEL [49,50,51] evaluates children and adolescents’ SES (self-awareness, self-regulation, social awareness, relationship skills, and responsible decision-making) using a 20 items scale (four items per domain). A 4-point Likert scale is used, ranging from 0 (never) to 3 (almost always), with a higher score meaning higher perceived social and emotional skills. Cronbach’s alpha reported acceptable to good internal consistency (self-awareness: α_pre_ = 0.77, α_post_ = 0.79; self-regulation: α_pre_ = 0.84, α_post_ = 0.86; social awareness: α_pre_ = 0.87, α_post_ = 0.87; relationship skills: α_pre_ = 0.79, α_post_ = 0.81; responsible decision making: α_pre_ = 0.89, α_post_ = 0.88).

*Academic outcomes*: It is composed of 3 single items that evaluate students’ academic motivation, engagement in learning, and academic performance. A 5-point Likert scale ranging from 0 (very poor) to 4 (excellent) is used, with a higher score meaning higher academic engagement in the three items.

*School level*: Participants were grouped by the different age grades of the two activities’ manuals developed in PROMEHS (k-4th: kindergarten to 4th grade; 5th–12th: 5th to 12th grade).

*Qualitative analysis*: During the implementation, teachers evaluated each activity they conducted in their class. In each, they had to indicate the theme of the activity selected [*Social and Emotional Learning (SEL)*, *Resilience* or *Behavioral, Social and Emotional Problems*], the method (online or onsite), as well as the adaptations made to the activities. Based on the first two indications, three variables were created: *type of implementation* (onsite, online, or mixed); the *number of activities implemented online* (sum of activities online); and *number of SEL activities implemented online* (sum of SEL activities online). The last indication teachers mentioned—adaptations for each activity—will be the subject of a qualitative analysis.

### 2.4. Data Analysis

Quantitative data analysis was performed using IBM SPSS Statistics (version 28.0). Missing data for the SDQ was between 0 and 0.4%; for SSIS-SEL, the percentage was between 0.2 and 1.1%; and for Academic Outcomes, between 0 and 0.1%. Thus, listwise deletion was used since missing data below 5% is inconsequential [47]. Cronbach’s alpha (α) was used to evaluate internal consistency, which was acceptable to good. 

A mixed between-within subjects analysis of variance (ANOVA) was conducted to assess the impact of the type of implementation used on the results across two time periods on students’ SDQ, SSIS-SEL, and Academic Outcomes scores. To assess the interaction effect, Wilks’s Lambda was reported. Only the onsite and mixed (online and onsite) implementation methodologies were used. Then, a one-way ANOVA was conducted to assess the impact of the number of online activities implemented on the domains evaluated. The dependent variables were computed for the difference between the post- and pre-test. The independent variable (number of activities) was grouped into two categories according to the number of activities implemented online (1: 1–3 activities; 2: 4–7 activities). Next, a mixed between-within subjects ANOVA was conducted on the same dependent variables to explore the differences between the number of activities performed online and by school level.

The SEL theme of PROMEHS was the only one where implementation occurred totally online, totally onsite, or mixed. Therefore, we conducted a mixed between-within subjects ANOVA to assess the impact of the methodology used on the results across two time periods on students’ scores of SSIS-SEL. The variable *number of SEL activities implemented* varied from 3 to 8. 

For these analyses, it was assured the assumptions were met. When Levene’s test for homogeneity of variants was significant, a more stringent significance value was defined (*p* < 0.001) for the mixed between-within subjects [53]. For the one-way ANOVA, when the assumption was violated, the Welch test was performed [53]. Since the sample size is reasonably similar, the ANOVA is reasonably robust to violations of the homogeneity of variance assumption [54]. Post-hoc comparisons were made using the Tukey test. Cohen’s *d* guidelines were used to interpret the effect sizes obtained [53], with small, medium, and large effects being 0.01, 0.06, and 0.14, respectively [55].

In the qualitative analysis, the MAXQDA 2022 software was used. Thematic analysis [56] included five steps according to LeCompte [57]: (1) tidying up with the organization of all data files in the software; (2) finding units of analysis by reading all files and identifying possible categories for the units; (3) creating stable sets of units of analysis; (4) creating patterns; and (5) assembling structures. 

## 3. Results

### 3.1. Comparison between Implementation Methodology (Onsite and Mixed)

A mixed between-within subjects ANOVA was conducted to assess the impact of onsite and type of implementation (type effect) on mental health (internalized problems, externalized problems, total of difficulties, and prosocial behavior), social and emotional competencies (SES: self-awareness, self-regulation, social awareness, relationship skills, and responsible decision-making), and academic outcomes (academic motivation, engagement in learning, and academic performance), across the two time periods.

The analysis did not reveal a significant interaction for type × time of implementation on any domains of mental health. There was a significant effect of time in all (see Table 1). There was a significant effect for the type of implementation, only for internalized problems [*F*(1, 931) = 3.95, *p* = 0.047, η_p_^2^ = 0.004)], although with a small effect. The onsite group had a smaller reduction of internalized problems than the mixed group, as presented in Table 1 (for more detailed information, see Table S1 in the Supplemental Material).

For SES, there was a significant type × time of implementation interaction on social awareness [*F*(1, 928) = 7.75, *p* = 0.005, η_p_^2^ = 0.008], relationship skills [*F*(1, 928) = 6.43, *p* = 0.011, η^2^ = 0.007], and responsible decision making [*F*(1, 928) = 5.09, *p* = 0.024, η_p_^2^ = 0.005], although will small effect sizes. There was an effect of time in all domains of social and emotional skills, as seen in Table 2. Additionally, there is an effect of type of implementation for relationship skills [*F*(1, 928) = 7.65, *p* = 0.006, η_p_^2^ = 0.008] and responsible decision-making [*F*(1, 928) = 4.94, *p* = 0.026, η_p_^2^ = 0.005], with the mixed group reporting higher levels on these domains compared to the onsite group. However, the effect sizes were small. For more detailed information, see Table S2 in the Supplemental Material.

For academic outcomes, there was a significant type × time of implementation interaction on academic performance [*F*(1, 930) = 7.19, *p* = 0.007, η_p_^2^ = 0.01] and a significant effect of time in all domains (see Table 3 or, for more detailed information, Table S3 in the Supplemental Material). In addition, there was a significant effect for the type of implementation in academic motivation [*F*(1, 930) = 6.36, *p* = 0.012, η_p_^2^ = 0.01] and academic achievement [*F*(1, 930) = 5.43, *p* = 0.020, η_p_^2^ = 0.006], with the onsite group having higher levels when compared to the mixed group.

### 3.2. Impact of the Number of Activities Implemented Online

As previously mentioned, a one-way ANOVA was conducted to evaluate the impact of the number of activities implemented online on mental health, social and emotional skills, and academic outcomes. For this analysis, we used the difference between both evaluation periods for each domain. Table 4, Table 5 and Table 6 present descriptive data, the *F* test for all the domains evaluated.

For all domains of mental health, there was a significant difference between groups, with group 1 (fewer online activities) having higher differences between evaluation periods, which means a higher reduction of problems and a higher increase in prosocial behavior (Table 4).

There was a significant difference between groups for self-awareness, social awareness, and relationship skills for SES domains, except for self-regulation and responsible decision-making. As shown in Table 5, group 1 shows higher differences between moments, indicating higher gains in social and emotional skills.

Regarding academic outcomes, no significant differences between groups were observed in all domains, which means that the differences in academic outcomes did not differ between the number of activities implemented.

### 3.3. Differences in Impact Considering the Number of Activities Online and School Level

A mixed between-within subjects ANOVA was conducted to explore the relation between the number of online activities and school level on the domains evaluated. The grouped variable, *number of activities online* (1: 1–3 activities; 2: 4–7 activities), was used, as well as the *school level* (k-4th: kindergarten to 4th grade; 5th–12th: 5th to 12th grade). Tables S4–S12 in the Supplemental Material present the means, standard deviations, *F* tests, and effect sizes.

On mental health domains, there was a significant interaction time × group × school level on externalized problems [*F*(1, 581) = 9.83, *p* = 0.002, η_p_^2^ = 0.02], and prosocial behavior [*F*(1, 581) = 8.86, *p* = 0.003, η_p_^2^ = 0.02], although with small effect sizes. In all domains, a significant interaction was observed on time x group of online activities and on time × school level. A significant effect of time, for *p* < 0.05, was also observed. In both the k-4th and 5th–12th groups, there is a higher reduction in group 1 of activities, i.e., the group with one to three online activities.

Between subjects, there was a significant interaction group × school level for internalized problems [*F*(1, 581) = 11.69, *p* < 0.001, η_p_^2^ = 0.02]. Regarding group effect, there was a significant difference in all domains. The effect of the school level was only significant for internalized problems, and K-4th had a higher reduction than 5th–12th.

On SES domains, there was a significant interaction for time × group × school level on self-regulation [*F*(1, 578) = 9.30, *p* = 0.002, η_p_^2^ = 0.02], social awareness [*F*(1, 578) = 17.20, *p* < 0.001, η_p_^2^ = 0.03], relationship skills [*F*(1, 578) = 10.77, *p* = 0.001, η_p_^2^ = 0.02], and responsible decision making [*F*(1, 578) = 11.11, *p* < 0.001, η_p_^2^ = 0.02]. There was a significant interaction for time × group for all domains except for self-regulation. Interaction time × school level and effect of time were significant for all SES domains. Except for the group with fewer online activities from 5th–12th, where a reduction of self-regulation was shown, all other domains increased at post-test. A higher increase in the group with fewer online activities in both school levels was observed for the other domains.

Between subjects, there was a significant interaction, group × school level, only for relationship skills [*F*(1, 578) = 4.09, *p* = 0.044, η_p_^2^ = 0.007]. Between groups (less online activities vs. more online activities), it was significant only for self-regulation; for school levels, it was significant for all, except for self-regulation and responsible decision-making. K-4th had a higher reduction in the SES domains compared to 5th–12th.

On academic outcomes, there was a significant interaction for time × group × school level for academic achievement [*F*(1, 580) = 5.55, *p* = 0.019, η_p_^2^ = 0.01]. No significant interaction time × group was observed, whereas a significant interaction time × school level and a main time effect was found for all domains. 

The interaction group × school level and the group effect were not significant between subjects. The school level effect was significant for all outcomes, with better results for k-4th.

### 3.4. SES Results Regarding Different Implementation Methods: Online, Onsite, and Mixed

A mixed between-within subjects ANOVA was conducted to assess the impact of the method used to implement SES activities (online, onsite, or mixed) on the students’ SSIS-SEL scores. For this analysis, only the cases with four SES activities were selected since it was the number requested for this theme in the project.

The analysis did report a significant type × time interaction for self-regulation [*F*(2, 667) = 3.73, *p* = 0.025, η_p_^2^ = 0.011]. Onsite implementation showed a lower mean at the pre-test and a higher mean at the post-test than mixed implementation. There was also a significant effect of time for all domains, as presented in Table 7. Type of implementation was significant in self-awareness [*F*(2, 667) = 3.02, *p* = 0.049, η_p_^2^ = 0.009], relationship skills [*F*(2, 667) = 4.34, *p* = 0.013, η_p_^2^ = 0.013], and responsible decision making [*F*(2, 667) = 3.44, *p* = 0.033, η_p_^2^ = 0.010]. Post-hoc comparisons using the Tukey test were used. For the domains where it was significant between groups, the online method had significantly higher means than the onsite group (see Table 7 or Tables S13 and S14 in the Supplemental Material).

### 3.5. Qualitative Analyses

Of the 35 teachers included in the dataset implemented in a mixed format, four were excluded for not mentioning digital adaptations, despite having implemented activities online. This analysis aimed to identify which adaptations teachers made when implementing activities online that included digital resources (e.g., platforms, game-apps, presentations, etc.). Two main categories were defined: synchronous (i.e., implemented during the online class schedule); and asynchronous activities (i.e., implemented outside the online class schedule). For each category, different subcategories were identified and will be presented below. For the present summary, the statements that are more representative of each category were selected (for more information regarding the number of subjects that mention each theme and subtheme, consult Table S15 in the Supplemental Material).

#### 3.5.1. Synchronous

Synchronous adaptations were the most used by teachers during online implementation. These adaptations for synchronous classes included: *digital platforms*, *presentations*, and *virtual classrooms*.

*Digital platforms* were the most indicated by the teachers (twenty teachers). These include platforms such as *Wordwall*, *Word Art*, *Mentimeter*, *Padlet*, *Poll Everywhere*, *Nearpod*, *VoKI*, *and Random Picker*, which teachers used to adapt the activity to online classes. Three teachers did not specify which platform was used.

“*Instead of writing on the board, they wrote on the chat, and afterward, it created a word cloud with the Word Art App.*” Teacher BF (3rd grade)

“*Presented the solutions wheel in the Worldwall app.*” Teacher LPR (7th grade).

“*The worksheet was transformed in Microsoft Forms with 33 questions and two hypotheses to choose from*”, and also, “*I created in the Padlet App a mural for students to share the diverse experiences they have lived.*” Teacher CS (10th grade).

*Presentations*. This subcategory includes presentations of videos (on YouTube), music, images, PowerPoint slides of the activity, or sharing the screen to present worksheets, and was mentioned by sixteen teachers.

“*I presented the story in PowerPoint, and the worksheet was also in the presentation, and I filled it out (as each child would identify their skills, talents, and passions).*” Teacher AS (5th grade).

“*Lastly, we talked about the changing hat—meaning I shared a picture of different umbrellas.*” Teacher CC (3rd grade).

“*The session began with the presentation of part of a music.*” Teacher RA (5th grade).

Six teachers mentioned virtual classrooms, mainly used as adaptations for group activities, where teachers defined groups and put the students in different virtual rooms.

“*The class was divided into two groups, and they were in two different virtual rooms to debate the consequences and strategies.*” Teacher MPB (4th grade).

“*Rooms in the TEAM Platform for a discussion in a more restricted group.*” Teacher CR (9th grade).

#### 3.5.2. Asynchronous

Asynchronous adaptations included the *execution of part of the activity* and *extra resources* where teachers sent more activities for students to do/see after.

*Execution of part of the activity before*, i.e., teachers sent part of the activity before the online class, so students prepared it in advance, was mentioned by seven teachers.

“*I used the asynchronous class. Students saw the video alone, at home, and answered the questions of the video exploration also at home, sending me their conclusions.*” Teacher PC (10th grade).

“*Parents were asked to share the video of the story.*” Teacher TF (Kindergarten).

“*Students wrote two individual qualities/characteristics and enumerated one quality/characteristic positive of each one of their classmates in a worksheet. Afterwards, they sent me the worksheet* via *e-mail.*” Teacher CC (3rd grade).

Lastly, three teachers sent extra resources for students.

“*In the Google Classroom Platform shared a cards game about emotions and breathing exercises.*” Teacher AA (4th grade).

“*Students watched three more YouTube videos in the asynchronous class.*” Teacher PC (10th grade).

## 4. Discussion

This study intended to investigate the impact of different implementation settings (online, mixed, and onsite) of a mental health curriculum at schools on students’ mental health indicators, social and emotional skills, and academic outcomes due to the restrictions imposed by the pandemic. It also aimed to evaluate the differences in impact considering the number of online activities implemented by itself and with school levels. Lastly, our qualitative analysis aimed to identify the online digital adaptations that teachers made to the curriculum, which were developed for onsite implementation. The findings from this study revealed that PROMEHS implementation during COVID-19 positively impacted students in both mixed and onsite implementation, despite more expressive results for the mixed format. Students who had fewer activities online showed greater improvements than those who had more online activities. When considering the school level, younger students (from kindergarten to 4th grade) had more positive results than older students (from 5th to 12th grade). When comparing the implementation format for SES activities, a totally online format reported better results, with an increase of SES at the post-test. Lastly, teachers reported different adaptations to implement the PROMEHS curriculum for the online format in synchronous (e.g., using digital platforms) and asynchronous (e.g., executing part of the activity before synchronous class) classes. The findings will be discussed in turn.

As mentioned, results showed that implementing the PROMEHS curriculum during COVID-19 positively impacted students, with the reduction of behavior problems and an increase in social and emotional skills and academic achievement, despite onsite or mixed implementation. Nevertheless, more expressive results were found in the mixed implementation on several domains, such as internalized problems, social awareness, relationship skills, and responsible decision-making. These results show that an online implementation, amid a lockdown period, was beneficial for children and adolescents. We can hypothesize that it acted as a protective factor since the literature on the impact of the lockdown reported an increase in mental health problems and academic achievement [11,14,58] and identified social and emotional skills, emotional regulation, and psychological resilience as protective factors [17,59]. Additionally, as reported in the study of Martinsone and colleagues [9], between evaluation moments that occurred during and after lockdown periods, students showed an increase of internalized and externalized problems and a reduction of social and emotional skills during COVID-19. Their study also found a significant negative correlation between adolescents’ social and emotional skills and internalizing and externalizing problems. 

Feelings of social disconnectedness and loneliness [13], lack of social support, absence of interactions with peers and teachers, and further interpersonal relationship problems [59,60,61,62] were concerns manifested by children and adolescents caused by school closures and the absence of their structural activities [59]. In this study, students that were part of the mixed group had the implementation of PROMEHS activities by their teachers during online classes. Even though the PROMEHS curriculum was developed to be implemented onsite, teachers adapted it to implement the activities during this difficult time. The activities part of the PROMEHS curriculum includes methodologies involving interactions between students and teachers, using teamwork, games, debates, and discussions. By fomenting these types of activities when many problems emerged, they were working towards reducing the impact of lockdown and also to lessen its effects on children and adolescents’ development [59,63].

Besides the impact on students’ mental health during lockdown periods, studies have also reported a reduction in students’ academic adjustment, achievement, performance, and motivation during the pandemic [15,64,65,66]. In the current study, after PROMEHS implementation, an increase in academic motivation and achievement was observed in both groups (mixed and onsite), with students from the mixed implementation group reporting higher academic achievement than those from the onsite group. Thus, it seems that PROMEHS positively impacted students’ mental health and social and emotional skills, which might have helped them cope better with the situation but also helped them with academic demands. Nevertheless, although PROMEHS also positively impacted academic motivation, it was less expressive for the mixed implementation. According to Klootwijk and colleagues [66], students had shown a decrease in academic motivation during online school compared to when school was onsite. Thus, students from the onsite group might have shown higher academic motivation when the implementation started, which could have been heightened by the PROMEHS curriculum, resulting in a more expressive increase than the mixed group.

It is important to consider that despite the adverse outcomes of lockdown on children and adolescents, some have thrived with it [14,61]. For example, a UK study that evaluated school students during the COVID-19 lockdown showed that those who reported improved mental wellbeing had lower levels of anxiety and depression, improved their relationship with others, felt less left out and lonely, and were less bullied [61]. When analyzing the results obtained in the current study, it is also important to consider that PROMEHS might positively impact those suffering from the lockdown and those who coped well. In this case, the PROMEHS program might be an extra support, adding more strategies to their repertoire.

When analyzing the mixed group, interesting results were found concerning the number of activities implemented online. The group with fewer online activities (one to three) showed better results than those with more online activities (four to seven). These results show that the PROMEHS curriculum helped students cope with the lockdown since it revealed an increase in SESs and a reduction of mental health problems, as previously indicated. Though, despite the adaptations performed by teachers for online implementation, the program had a higher impact when activities were mostly performed onsite.

In our sample, children and adolescents reduced their internalized and externalized problems and increased their prosocial behavior with PROMEHS implementation. Nevertheless, it was higher in the groups with mixed implementation, specifically for those with fewer online activities. In accordance with the results from the study of Liu and colleagues [67], that the school reopening might reduce by itself the prevalence of internalized problems, we can hypothesize that by having online implementation which is maintained when transitioning by having the implementation of the activities in onsite settings, it can contribute to a higher reduction of these problems. On the contrary, if children increase the expression of emotional and behavioral problems and reduce prosocial behavior, as found by Wang and colleagues [68], the continuity of the implementation can serve as relevant support regarding the needs that children and adolescents might show [24,45].

Regarding the school level, we found that students from kindergarten to 4th grade had better results than those from 5th to 12th grade, with a higher reduction of mental health problems and increased social and emotional skills and academic outcomes. These results (except for self-regulation in older students) might be related to a higher impact of lockdown on adolescents, who suffered more compared to children, and therefore had higher impacts on their mental health [11,14,58], or to the program itself since, according to Yeager [69], these programs tend to show more effectiveness in children

For SEL activities, when comparing the three types of implementations (online, onsite, and mixed), all revealed positive results. There was a significant difference in self-awareness, relationship skills, and responsible decision-making between online and onsite settings. The first reported better results but no differences between groups for self-regulation and social awareness. Again, these findings show the importance of PROMEHS implementation during and after the lockdown.

Considering previous studies on the impact of the pandemic [10,11,12,13], our study shows the importance of implementing programs that promote the mental health of children and adolescents to counteract the impacts of the pandemic. 

The results of this study are consistent with previous studies on the impact of mental health programs implemented at schools, which report an increase in social and emotional skills, academic achievement, and a reduction of behavioral problems [19,20,70]. It is also consistent with the results from the study by Li and colleagues [43], which reported increases in social and emotional skills with the adaptation of an SEL program for online learning during COVID-19. In addition, our results align with the study of Cefai and colleagues, which compiles data from the six European countries where PROMEHS was implemented, including Portugal [46]. Nevertheless, it was not possible to evaluate the impact of a total online implementation for the whole implementation period or consider the various PROMEHS themes. 

The results from the qualitative analysis reported that teachers could adapt the program to an online setting using their digital skills. Considering that the implementation did not occur during the first lockdown, teachers were already familiarized and had more knowledge about online learning, which could have facilitated their ability to adapt the activities. Adaptations occurred mainly in synchronous classes using platforms such as Wordwall, Nearpod, Padlet, and others. They also used video, pictures, and PowerPoint presentations and used virtual classes to enable teamwork between groups. Asynchronous adaptations included the execution of part of the activity and extra resources. These adaptations from teachers align with those suggested by Currie and colleagues [42] for the online delivery of social and emotional skills programs, and Chen and Adams [44].

The present study has limitations and strengths. Regarding limitations, it was not possible to evaluate a total online implementation. Further, the pre-test was conducted before and during the lockdown, which can produce bias regarding the results. Teachers might perceive students differently when evaluating them before or during the lockdown. Lastly, there was no information about students’ age, and for that reason, school-level groups were created. 

This study adds knowledge regarding the impact of interventions during the pandemic, answering the United Nations’ solicitation on evaluating interventions conducted during this critical period [2]. Second, this study evaluates social and emotional skills, a domain that, to our knowledge, is lacking in evaluation during the pandemic. Third, this study had participants from urban and rural areas from different regions in Portugal, which allowed us to have a more global evaluation of the program’s impact in the country. Fourth, the sample comprises students from different school levels, being evaluated in groups using the school level variable. Lastly, this study identified adaptations of mental health programs to an online context made by teachers, which can help professionals develop or adapt programs.

Further investigation is needed regarding the impact of mental health and wellbeing programs at schools, which can be helpful for other periods of crisis, namely other pandemics or for situations that demand online settings (e.g., lockdown due to health risks). Additionally, the literature on the impact of the lockdown on children and adolescents’ social and emotional skills, as well as the mental health of this population after lockdown, is scarce, limiting the discussion. It is important to consider more rigorous adaptations when adapting onsite programs to online and designing programs specifically for online settings. These adaptations should consider digital competencies and the reality/needs of the school and family contexts.

## 5. Conclusions

Answering the question presented in the title *Does online implementation make a difference in the effects of a mental health curriculum at schools?*, we can say that it does make a difference in students’ SES, mental health, and academic outcomes. Compared to those who had an exclusive implementation onsite, results show that those who started the implementation online benefited more, with a more expressive and significant reduction of internalized problems and an increase in relationship skills, responsible decision making and academic achievement. Students who had a total online implementation of SEL activities reported higher self-awareness, relationship skills, and responsible decision-making.

However, despite these differences between implementation settings, all students benefited from the implementation of PROMEHS, regardless of the type of implementation, which reveals that with proper adaptations, it is possible to implement these programs during online school, not limited to the onsite setting. The results of this study also reinforce the already extensive research on mental health programs implemented at schools. 

When focusing on the mixed implementation, those with fewer activities online reported decreased mental health problems and increased SES and academic outcomes compared to those with more activities online. In addition, younger students from kindergarten to 4th grade had more expressive results in all domains than older students from 5th to 12th grade.

Teachers that conducted part of the implementation online were able to adapt the PROMEHS curriculum to this setting, using both synchronous and asynchronous adaptions. Synchronous adaptations focused on the use of digital platforms, the presentation of videos, music and pictures, and virtual rooms to transform the activity initially developed for onsite implementation. The limitations of online classes were related to the time per class, which made teachers proceed with the execution of part of the activity in asynchronous classes. 

Finally, implementing this program amid a lockdown was positive and important in reducing its impact on children and adolescents, with social skills being promoted and mental health problems reduced. Therefore, we can also conclude that implementing mental health programs at school during the pandemic can be an important protective factor and can be used as a way to counteract its impact on children and adolescents.

## Figures and Tables

**Table 1:** Mental health for mixed and onsite implementation groups across the two time periods. *p*-values for interaction (type × time), time, and type effects.

	Onsite					Mixed					*p*		
		Pre		Post			Pre		Post		Type Effect	Time Effect	Type × Time Interaction
	*n*	*M*	*SD*	*M*	*SD*	*n*	*M*	*SD*	*M*	*SD*			
Internalized Problems	348	0.42	0.35	0.35	0.34	585	0.39	0.34	0.30	0.33	**0.047**	**<0.001**	0.243
Externalized Problems		0.53	0.43	0.47	0.42		0.49	0.44	0.46	0.43	0.402	**<0.001**	0.155
Prosocial Behavior		1.58	0.47	1.60	0.45		1.52	0.48	1.59	0.46	0.195	**<0.001**	0.079

**Table 2:** SES for mixed and onsite groups across the two time periods. *p*-values for interaction (Type × Time), time, and type effects.

	Onsite					Mixed					*p*		
		Pre		Post			Pre		Post		Type Effect	Time Effect	Type × Time Interaction
	*n*	*M*	*SD*	*M*	*SD*	*n*	*M*	*SD*	*M*	*SD*			
Self-awareness	348	2.93	0.57	3.07	0.51	582	2.98	0.64	3.17	0.62	0.051	**<0.001**	0.112
Self-regulation		2.96	0.63	3.04	0.66		2.99	0.72	3.08	0.70	0.373	**<0.001**	0.806
Social Awareness		3.22	0.61	3.26	0.55		3.19	0.63	3.34	0.59	0.459	**<0.001**	**0.005**
Interpersonal relationships		3.19	0.54	3.27	0.51		3.25	0.58	3.41	0.58	**0.006**	**<0.001**	**0.011**
Responsible Decision Making		3.23	0.61	3.29	0.58		3.28	0.65	3.42	0.63	**0.026**	**<0.001**	**0.024**

**Table 3:** Academic Outcomes for mixed and onsite groups across the two time periods. Mean values, standard deviations, and *p*-values for interaction (type × time), time, and type effects.

	Onsite					Mixed					*p*		
		Pre		Post			Pre		Post		Type Effect	Time Effect	Type × Time Interaction
	*n*	*M*	*SD*	*M*	*SD*	*n*	*M*	*SD*	*M*	*SD*			
Academic Motivation	348	3.83	1.01	3.92	1.08	584	3.68	0.97	3.75	1.01	**0.012**	**0.001**	0.676
Engagement in Learning		3.79	1.04	3.85	1.02		3.66	0.96	3.76	1.01	0.070	**0.001**	0.465
Academic Achievement		3.75	1.01	3.82	1.01		3.55	0.89	3.74	0.96	**0.020**	**<0.001**	**0.007**

**Table 4:** Mean differences between pre- and post-test, standard deviations, and *F* tests for the mental health domains within each group.

Domains	Group	*M*	*SD*	*F* Test
Internalized Problems	1	−0.13	0.35	*F*(1, 450.51) = 11.27, ***p* < 0.001** ^a^
	2	−0.05	0.26	
Externalized Problems	1	−0.08	0.33	*F*(1, 462.26) = 11.74, ***p* < 0.001** ^a^
	2	0.01	0.25	
Prosocial Behavior	1	0.12	0.45	*F*(1, 472.62) = 6.79, ***p* = 0.009** ^a^
	2	0.03	0.36	

Note: 1: 1 to 3 activities implemented online; 2: 4 to 7 activities implemented online; ^a^ Welch’s *F* test conducted.

**Table 5:** Mean values, standard deviations, and *F* tests for SES domains within each group.

Domains	Group	*M*	*SD*	*F* Test
Self-awareness	1	0.27	0.64	*F*(1, 448.97) = 7.37, ***p* = 0.007** ^a^
	2	0.14	0.47	
Self-regulation	1	0.10	0.56	*F*(1, 467.95) = 0.49, *p* = 0.483 ^a^
	2	0.07	0.44	
Social Awareness	1	0.22	0.62	*F*(1, 436.72) = 8.89, ***p =* 0.003** ^a^
	2	0.08	0.44	
Relationship Skills	1	0.23	0.55	*F*(1, 580) = 9.14, ***p =* 0.003**
	2	0.10	0.45	
Responsible Decision Making	1	0.18	0.61	*F*(1, 580) = 2.89, *p = 0*.090
	2	0.11	0.51	

Note: 1: 1 to 3 activities implemented online; 2: 4 to 7 activities implemented online; ^a^ Welch’s *F* test conducted.

**Table 6:** Mean values, standard deviations, and *F* tests for academic outcomes within each group.

Domains	Group	*M*	*SD*	*F* Test
Academic Motivation	1	0.07	0.77	*F*(1, 582) = 0.00, *p* = 0.970
	2	0.07	0.72	
Engagement in Learning	1	0.11	0.75	*F*(1, 582) = 0.05, *p =* 0.831
	2	0.09	0.72	
Academic Achievement	1	0.22	0.75	*F*(1, 582) = 0.51, *p* = 0.477
	2	0.18	0.70	

Note: 1: 1 to 3 activities implemented online; 2: 4 to 7 activities implemented online.

**Table 7:** SES domains across the two time periods, considering the method. *p*-values for interaction (type × time), time, and type effects.

	Online					Onsite					Mixed					*p*		
		Pre		Post			Pre		Post			Pre		Post		Type Effect	Time Effect	Type × Time Interaction
	*n*	*M*	*SD*	*M*	*SD*	*n*	*M*	*SD*	*M*	*SD*	*n*	*M*	*SD*	*M*	*SD*			
Self-awareness	299	3.04	0.62	3.17	0.61	144	2.89	0.64	3.03	0.59	227	2.99	0.63	3.11	0.63	**0.049**	**<0.001**	0.895
Self-regulation		3.05	0.66	3.14	0.67		2.91	0.70	3.06	0.71		2.97	0.79	2.99	0.76	0.089	**<0.001**	**0.025**
Social Awareness		3.25	0.56	3.32	0.60		3.19	0.67	3.25	0.61		3.25	0.63	3.31	0.58	0.518	**<0.001**	0.946
Relationship Skills		3.32	0.55	3.42	0.58		3.17	0.61	3.33	0.58		3.25	0.57	3.33	0.58	**0.013**	**<0.001**	0.843
Responsible Decision Making		3.33	0.59	3.44	0.63		3.19	0.68	3.27	0.67		3.31	0.68	3.36	0.64	**0.033**	**<0.001**	0.431

## Data Availability

The data presented in this study are available upon reasonable request from the corresponding author.

## Supplementary Materials

The following supporting information can be downloaded at: https://www.mdpi.com/article/10.3390/ijerph192416990/s1, Table S1: Mental Health for mixed and onsite implementation groups across the two time periods. P-values for interaction (type × time), time and type effects; Table S2: SES for mixed and onsite groups across the two time periods. *F tests* and *effect sizes* for interaction (type × time), time and type effects; Table S3: Academic Outcomes for mixed and onsite groups across the two time periods. Mean values, standard deviations, *F tests,* and *effect sizes* for interaction (type × time), time and type effects; Table S4: Mental Health for School Level and the number of online activities across the two time periods. Mean values, standard deviations; Table S5: Mental Health for School Level and the number of online activities across the two time periods. *F tests* and *effect sizes* for interaction within subjects; Table S6: Mental Health for School Level and the number of online activities across the two time periods. *F tests* and *effect sizes* for interaction between subjects; Table S7: SES for School Level and the number of online activities across the two time periods. Mean values, standard deviations; Table S8: SES for School Level and the number of online activities across the two time periods. *F tests* and *effect sizes* for interaction within subjects; Table S9: SES for School Level and the number of online activities across the two time periods. *F tests* and *effect sizes* for interaction between subjects; Table S10: Academic Outcomes for School Level and the number of online activities across the two time periods. Mean values, standard deviations; Table S11: Academic Outcomes for School Level and the number of online activities across the two time periods. *F tests* and *effect sizes* for interaction within subjects; Table S12: Academic Outcomes for School Level and the number of online activities across the two time periods. *F tests* and *effect sizes* for interaction between subjects; Table S13: SES across the two time periods considering the methodology. Means and standard deviations; Table S14: SES domains across the two time periods considering the methodology. *F tests and effect sizes*, for interaction (type × time), time and type effects; Table S15: ‘Teachers’ adaptations for online activities using digital resources (Number of Subjects that mention each theme and subtheme).
