# Supplementary material for: Does Online Implementation Make a Difference in the Effects of a Mental Health Curriculum at Schools?
Source: Int J Environ Res Public Health. 2022 Dec 17;19(24):16990. doi: 10.3390/ijerph192416990 (PMC9779368; doi:10.3390/ijerph192416990)
Supplement: Supplementary file 1 [file ijerph-19-16990-s001.zip › ijerph-2095168-supplementary.pdf]

Table S1. Mental Health for mixed and onsite implementation groups across the two time periods. P-values for interaction (Type x Time), time, and type effects.

	**Onsite**					**Mixed**					***F test, effect size***		
		**Pre**		**Post**			**Pre**		**Post**		**Type effects**	**Time effect**	**Type x Time Interaction**
	n	M	SD	M	SD	n	M	SD	M	SD			
Internalized Problems		0.42	0.35	0.35	0.34		0.39	0.34	0.30	0.33	$F(1, 931) = 3.95$, **$p = 0.047$**, $\eta_p^2 = 0.004$	$F(1, 931) = 53.32$, **$p < 0.001$**, $\eta_p^2 = 0.054$	$F(1, 931) = 1.37$, $p = 0.243$, $\eta_p^2 = 0.001$
Externalized Problems	348	0.53	0.43	0.47	0.42	585	0.49	0.44	0.46	0.43	$F(1, 931) = 0.70$, $p = 0.402$, $\eta_p^2 = 0.001$	$F(1, 931) = 19.96$, **$p < 0.001$**, $\eta_p^2 = 0.021$	$F(1, 931) = 2.03$, $p = 0.155$, $\eta_p^2 = 0.002$
Prosocial Behavior		1.58	0.47	1.60	0.45		1.52	0.48	1.59	0.46	$F(1, 931) = 1.68$, $p = 0.195$, $\eta_p^2 = 0.002$	$F(1, 931) = 11.45$, **$p < 0.001$**, $\eta_p^2 = 0.012$	$F(1, 931) = 3.09$, $p = 0.079$, $\eta_p^2 = 0.003$

Table S2. SES for mixed and onsite groups across the two time periods. *F tests* and *effect sizes* for interaction (Type x Time), time, and type effects.

	**Onsite**					**Mixed**					***F test, effect size***		
		**Pre**		**Post**			**Pre**		**Post**		**Type effect**	**Time effect**	**Type x Time Interaction**
	n	M	SD	M	SD	n	M	SD	M	SD			
Self-awareness		2.93	0.57	3.07	0.51		2.98	0.64	3.17	0.62	$F(1, 928) = 3.83$, $p = 0.051$, $\eta_p^2 = 0.004$	$F(1, 928) = 84.65$, **$p < 0.001$**, $\eta_p^2 = 0.084$	$F(1, 928) = 2.53$, $p = 0.112$, $\eta_p^2 = 0.003$
Self-regulation		2.96	0.63	3.04	0.66		2.99	0.72	3.08	0.70	$F(1, 928) = 0.80$, $p = 0.373$, $\eta_p^2 = 0.001$	$F(1, 928) = 25.43$, **$p < 0.001$**, $\eta_p^2 = 0.027$	$F(1, 928) = 0.06$, p **= 0.**806, $\eta_p^2 = 0.000$
	348					582							
Social Awareness		3.22	0.61	3.26	0.55		3.19	0.63	3.34	0.59	$F(1, 928) = 0.55$, $p = 0.459$, $\eta_p^2 = 0.001$	$F(1, 928) = 27.52$, **$p < 0.001$**, $\eta_p^2 = 0.029$	$F(1, 928) = 7.75$, **$p = 0.005$**, $\eta_p^2 = 0.008$
Relationship Skills		3.19	0.54	3.27	0.51		3.25	0.58	3.41	0.58	$F(1, 928) = 7.65$, **$p = 0.006$**, $\eta_p^2 = 0.008$	$F(1, 928) = 52.34$, **$p < 0.001$**, $\eta_p^2 = 0.053$	$F(1, 928) = 6.43$, **$p = 0.011$**, $\eta_p^2 = 0.007$

	n	M	SD	M	SD	n	M	SD	M	SD	Type effect	Time effect	Type x Time Interaction
Responsible Decision Making		3.23	0.61	3.29	0.58		3.28	0.65	3.42	0.63	$F(1, 928) = 4.94$, $\boldsymbol{p = 0.026}$, $\eta_p^2 = 0.005$	$F(1, 928) = 31.09$, $\boldsymbol{p < 0.001}$, $\eta_p^2 = 0.032$	$F(1, 928) = 5.09$, $\boldsymbol{p = 0.024}$, $\eta_p^2 = 0.005$

Table S3. Academic Outcomes for mixed and onsite groups across the two time periods. Mean values, Standard Deviations, *F tests*, and *effect sizes* for interaction (Type x Time), time, and type effects.

	**Onsite**					**Mixed**					***F test, effect size***		
		**Pre**		**Post**			**Pre**		**Post**		**Type effect**	**Time effect**	**Type x Time Interaction**
	n	M	SD	M	SD	n	M	SD	M	SD			
Academic Motivation		3.83	1.01	3.92	1.08		3.68	0.97	3.75	1.01	$F(1, 930) = 6.36$, $\boldsymbol{p = 0.012}$, $\eta_p^2 = 0.007$	$F(1, 930) = 10.25$, $\boldsymbol{p = 0.001}$, $\eta_p^2 = 0.011$	$F(1, 930) = 0.18$, $p = 0.676$, $\eta_p^2 = 0.000$
Engagement in Learning	348	3.79	1.04	3.85	1.02	584	3.66	0.96	3.76	1.01	$F(1, 930) = 3.29$, $p = 0.070$, $\eta_p^2 = 0.004$	$F(1, 930) = 10.83$, $\boldsymbol{p = 0.001}$, $\eta_p^2 = 0.012$	$F(1, 930) = 0.53$, $p = 0.465$, $\eta_p^2 = 0.001$
Academic Achievement		3.75	1.01	3.82	1.01		3.55	0.89	3.74	0.96	$F(1, 930) = 5.43$, $\boldsymbol{p = 0.020}$, $\eta_p^2 = 0.006$	$F(1, 930) = 33.17$, $\boldsymbol{p < 0.001}$, $\eta_p^2 = 0.034$	$F(1, 930) = 7.19$ $\boldsymbol{p = 0.007}$, $\eta_p^2 = 0.008$

Table S4. Mental Health for School Level and the number of online activities across the two time periods. Mean values, Standard Deviations.

	K – 4th										5th – 12th									
	1					2					1					2				
		Pre		Post			Pre		Post			Pre		Post			Pre		Post	
	n	M	SD	M	SD	n	M	SD	M	SD	n	M	SD	M	SD	n	M	SD	M	SD
Internalized Problems		0.48	0.35	0.29	0.31		0.28	0.24	0.22	0.24		0.40	0.37	0.31	0.34		0.40	0.35	0.36	0.35
Externalized Problems	115	0.65	0.45	0.49	0.40	143	0.47	0.39	0.47	0.43	138	0.50	0.48	0.49	0.48	189	0.41	0.41	0.42	0.42
Prosocial Behavior		1.50	0.49	1.74	0.34		1.52	0.48	1.57	0.47		1.62	0.45	1.64	0.45		1.45	0.49	1.47	0.49

Table S5. Mental Health for School Level and the number of online activities across the two time periods. *F tests* and *effect sizes* for interaction within subjects.

	F test* and *effect size Within Subjects			
	**Time effect**	**Time x Group**	**Time x School Level**	**Time x Group x School Level**
Internalized Problems	$F(1, 581) = 56.44$, p **< 0.001**, $\eta_p^2 = 0.089$	$F(1, 581) = 13.02$, p **< 0.001,** $\eta_p^2 = 0.022$	$F(1, 581) = 5.75$, p = 0.**017,** $\eta_p^2 = 0.010$	$F(1, 581) = 2.61$, $p = 0.107$, $\eta_p^2 = 0.004$
Externalized Problems	$F(1, 581) = 10.35$, p = 0.**001,** $\eta_p^2 = 0.018$	$F(1, 581) = 14.81$, p **< 0.001,** $\eta_p^2 = 0.025$	$F(1, 581) = 11.87$, p **< 0.001,** $\eta_p^2 = 0.020$	$F(1, 581) = 9.83$, p = 0.**002,** $\eta_p^2 = 0.017$
Prosocial Behavior	$F(1, 581) = 24.25$, p **< 0.001,** $\eta_p^2 = 0.040$	$F(1, 581) = 8.76$, p = 0.**003,** $\eta_p^2 = 0.015$	$F(1, 581) = 13.94$, ***p* < 0.001,** $\eta_p^2 = 0.023$	$F(1, 581) = 8.86$, ***p* =** 0.**003,** $\eta_p^2 = 0.015$

Table S6. Mental Health for School Level and the number of online activities across the two time periods. *F tests* and *effect sizes* for interaction between subjects.

	F test* and *effect size Between Subjects		
	**Group effect**	**School Level**	**Group x School Level**
Internalized Problems	*F*(1, 581) = 4.97, ***p* = 0.026,** η_p^2= 0.008	*F*(1, 581) = 5.01**, *p* = 0.026,** η_p^2= 0.009	*F*(1, 581) = 11.68, ***p* < 0.001,** η_p^2= 0.020
Externalized Problems	*F*(1, 581) = 6.33, *p* = 0.**012,** η_p^2= 0.011	*F*(1, 581) = 3.56, *p* = 0.060**,** η_p^2= 0.006	*F*(1, 581) = 0.11, *p* = 0.741**,** η_p^2= 0.000
Prosocial Behavior	*F*(1, 581) = 12.67, *p* **< 0.001,** η_p^2= 0.021	*F*(1, 581) = 1.01, *p* = 0.317**,** η_p^2= 0.002	*F*(1, 581) = 2.08, *p* = 0.150**,** η_p^2= 0.004

Table S7. SES for School Level and the number of online activities across the two time periods. Mean values, Standard Deviations.

	**K – 4th**										**5th – 12th**									
	**1**					**2**					**1**					**2**				
		**Pre**		**Post**			**Pre**		**Post**			**Pre**		**Post**			**Pre**		**Post**	
	n	M	SD	M	SD	n	M	SD	M	SD	n	M	SD	M	SD	n	M	SD	M	SD
Self-awareness		2.86	0.67	3.36	0.53		3.05	0.63	3.33	0.53		2.99	0.65	3.06	0.66		2.98	0.63	3.01	0.63
Self-regulation		2.82	0.73	3.13	0.64		3.00	0.66	3.15	0.62		2.99	0.84	2.92	0.79		3.09	0.65	3.11	0.71
Social Awareness	115	3.05	0.66	3.52	0.52	141	3.29	0.63	3.43	0.57	138	3.25	0.69	3.27	0.62	188	3.17	0.55	3.21	0.61
Relationship Skills		3.16	0.57	3.57	0.49		3.42	0.56	3.55	0.55		3.24	0.59	3.32	0.61		3.19	0.57	3.27	0.58
Responsible Decision Making		3.11	0.72	3.50	0.53		3.32	0.63	3.47	0.61		3.33	0.71	3.35	0.68		3.31	0.57	3.39	0.64

Table S8. SES for School Level and the number of online activities across the two time periods. *F tests* and *effect sizes* for interaction within subjects.

	F test **and** *effect size Within Subjects*			
	**Time effect**	**Time x Group**	**Time x School Level**	**Time x Group x School Level**
Self-awareness	$F(1, 578) = 99.19$, **$p < 0.001$,** $\eta_p^2 = 0.146$	$F(1, 578) = 8.77$, p = 0.**003,** $\eta_p^2 = 0.015$	$F(1, 578) = 58.06$**, $p < 0.001$,** $\eta_p^2 = 0.091$	$F(1, 578) = 3.70$, $p = 0.055$, $\eta_p^2 = 0.006$
Self-regulation	$F(1, 578) = 24.97$, **$p < 0.001$,** η_p^2 0.041	$F(1, 578) = 0.88$, $p = 0.349$, $\eta_p^2 = 0.002$	$F(1, 578) = 39.12$, **$p < 0.001$,** $\eta_p^2 = 0.063$	$F(1, 578) = 9.30$, **p =** 0.**002,** $\eta_p^2 = 0.016$
Social Awareness	$F(1, 578) = 58.13$, **$p < 0.001$,** $\eta_p^2 = 0.091$	$F(1, 578) = 12.53$, p **< 0.001,** $\eta_p^2 = 0.021$	$F(1, 578) = 39.47$, **$p < 0.001$,** $\eta_p^2 = 0.064$	$F(1, 578) = 17.20$, **p <** 0.**001,** $\eta_p^2 = 0.029$
Relationship Skills	$F(1, 578) = 73.11$, **$p < 0.001$,** $\eta_p^2 = 0.112$	$F(1, 578) = 11.13$, p **< 0.001,** $\eta_p^2 = 0.019$	$F(1, 578) = 19.87$, **$p < 0.001$,** $\eta_p^2 = 0.033$	$F(1, 578) = 10.77$, **p =** 0.**001,** $\eta_p^2 = 0.018$
Responsible Decision Making	$F(1, 578) = 46.17$, **$p < 0.001$,** $\eta_p^2 = 0.074$	$F(1, 578) = 3.98$, **$p = 0.047$,** $\eta_p^2 = 0.007$	$F(1, 578) = 21.96$, **$p < 0.001$,** $\eta_p^2 = 0.037$	$F(1, 578) = 11.11$, **$p < 0.001$,** $\eta_p^2 = 0.019$

Table S9. SES for School Level and the number of online activities across the two time periods. *F tests* and *effect sizes* for interaction between subjects.

	F test **and** *effect size Between Subjects*		
	**Group effect**	**School Level effect**	**Group x School Level**
Self-awareness	$F(1, 578) = 0.210$, $p = 0.625$**,** $\eta_p^2 = 0.000$	$F(1, 578) = 8.40$, p = 0.**004,** $\eta_p^2 = 0.014$	$F(1, 578) = 1.30$, $p = 0.255$, $\eta_p^2 = 0.002$
Self-regulation	$F(1, 578) = 4.63$, **p =** 0.**032,** $\eta_p^2 = 0.008$	$F(1, 578) = 0.00$, $p = 0.972$**,** $\eta_p^2 = 0.000$	$F(1, 578) = 0.10$, $p = 0.758$**,** $\eta_p^2 = 0.000$
Social Awareness	$F(1, 578) = 0.00$, $p = 0.947$, $\eta_p^2 = 0.000$	$F(1, 578) = 4.65$, **$p = 0.031$,** $\eta_p^2 = 0.008$	$F(1, 578) = 2.46$, $p = 0.118$, $\eta_p^2 = 0.004$
Relationship Skills	$F(1, 578) = 0.60$, $p = 0.438$, $\eta_p^2 = 0.001$	$F(1, 578) = 15.47$, **$p < 0.001$,** $\eta_p^2 = 0.026$	$F(1, 578) = 4.09$, **p =** 0.**044,** $\eta_p^2 = 0.007$
Responsible Decision Making	$F(1, 578) = 1.12$, $p = 0.290$**,** $\eta_p^2 = 0.002$	$F(1, 578) = 0.01$, $p = 0.925$**,** $\eta_p^2 = 0.000$	$F(1, 578) = 0.71$, $p = 0.401$**,** $\eta_p^2 = 0.001$

Table S10. Academic Outcomes for School Level and the number of online activities across the two time periods. Mean values, Standard Deviations.

	**K – 4th**										**5th – 12th**									
	**1**					**2**					**1**					**2**				
		**Pre**		**Post**			**Pre**		**Post**			**Pre**		**Post**			**Pre**		**Post**	
	n	M	SD	M	SD	n	M	SD	M	SD	n	M	SD	M	SD	n	M	SD	M	SD
Academic Motivation		3.81	0.99	4.07	0.94		3.77	0.85	3.95	0.95		3.72	1.13	3.62	1.01		3.52	0.91	3.51	1.01
Engagement in Learning	115	3.85	0.92	4.08	0.94	143	3.78	0.85	3.93	0.98	138	3.65	1.05	3.66	0.98	188	3.45	0.96	3.50	1.03
Academic Achievement		3.65	0.97	4.10	0.91		3.66	0.86	3.92	0.93		3.51	0.93	3.54	0.91		3.41	0.83	3.53	0.97

	F test and effect size Within Subjects			
	**Time effect**	**Time x Group**	**Time x School Level**	**Time x Group x School Level**
Academic Motivation	*F*(1, 580) = 7.37, ***p* = 0.007**, η_p^2= 0.013	*F*(1, 580) = 0.00, *p* = 0.995, η_p^2= 0.000	*F*(1, 580) = 20.36**,** ***p* < 0.001,** η_p^2= 0.034	*F*(1, 578) = 1.65, *p* = 0.200**,** η_p^2= 0.003
Engagement in Learning	*F*(1, 580) = 12.50, ***p* < 0.001,** η_p^2= 0.021	*F*(1, 580) = 0.07, *p* = 0.786**,** η_p^2= 0.000	*F*(1, 580) = 6.50, ***p* = 0.011,** η_p^2= 0.011	*F*(1, 580) = 1.04, *p* = 0.308**,** η_p^2= 0.002
Academic Achievement	*F*(1, 580) = 51.41, ***p* < 0.001,** η_p^2= 0.081	*F*(1, 580) = 0.78, *p* **= 0**.378**,** η_p^2= 0.001	*F*(1, 580) = 22.34, ***p* < 0.001,** η_p^2= 0.037	*F*(1, 580) = 5.55, ***p* =** 0.**019,** η_p^2= 0.009

Table S11. Academic Outcomes for School Level and the number of online activities across the two time periods. *F tests* and *effect sizes* for interaction within subjects.

	F test and effect size Between Subjects		
	**Group effect**	**School Level effect**	**Group x School Level**
Academic Motivation	$F(1, 580) = 2.41$, $p = 0.121$, $\eta_p^2 = 0.004$	$F(1, 580) = 16.39$, $\boldsymbol{p < 0.001}$, $\eta_p^2 = 0.027$	$F(1, 580) = 0.26$, $p = 0.608$, $\eta_p^2 = 0.000$
Engagement in Learning	$F(1, 580) = 3.73$, $p = 0.054$, $\eta_p^2 = 0.006$	$F(1, 580) = 21.14$, $\boldsymbol{p < 0.001}$, $\eta_p^2 = 0.035$	$F(1, 580) = 0.24$, $p = 0.624$, $\eta_p^2 = 0.000$
Academic Achievement	$F(1, 580) = 0.99$, $p = 0.320$, $\eta_p^2 = 0.002$	$F(1, 580) = 22.57$, $\boldsymbol{p < 0.001}$, $\eta_p^2 = 0.037$	$F(1, 580) = 0.04$, $p = 0.837$, $\eta_p^2 = 0.000$

Table S12. Academic Outcomes for School Level and the number of online activities across the two time periods. *F tests* and *effect sizes* for interaction between subjects.

Table S13. SES across the two time periods considering the methodology. Means and Standard Deviations.

	**Online**					**Onsite**					**Mixed**				
		**Pre**		**Post**			**Pre**		**Post**			**Pre**		**Post**	
	n	M	SD	M	SD	n	M	SD	M	SD	n	M	SD	M	SD
Self-awareness	299	3.04	0.62	3.17	0.61	144	2.89	0.64	3.03	0.59	227	2.99	0.63	3.11	0.63
Self-regulation		3.05	0.66	3.14	0.67		2.91	0.70	3.06	0.71		2.97	0.79	2.99	0.76
Social Awareness		3.25	0.56	3.32	0.60		3.19	0.67	3.25	0.61		3.25	0.63	3.31	0.58
Relationship Skills		3.32	0.55	3.42	0.58		3.17	0.61	3.33	0.58		3.25	0.57	3.33	0.58

Responsible Decision Making	3.33	0.59	3.44	0.63		3.19	0.68	3.27	0.67		3.31	0.68	3.36	0.64

Table S14. SES across the two time periods considering the methodology. *F tests* and *effect sizes* for interaction (Type x Time), time, and type effects.

	F test, effect size		
	**Type effect**	**Time effect**	**Type x Time Interaction**
Self-awareness	$F(2, 667) = 3.02$, $\boldsymbol{p = 0.049}$**,** $\eta_p^2 = 0.009$	$F(2, 667) = 40.40$, $\boldsymbol{p < 0.001}$**,** $\eta_p^2 = 0.057$	$F(2, 667) = 0.11$, $p = 0.895$, $\eta_p^2 = 0.000$
Self-regulation	$F(2, 667) = 2.43$, $p = 0.089$, $\eta_p^2 = 0.007$	$F(2, 667) = 19.04$, $\boldsymbol{p < 0.001}$**,** $\eta_p^2 = 0.028$	$F(2, 667) = 3.73$, $\boldsymbol{p = 0.025}$**,** $\eta_p^2 = 0.011$
Social Awareness	$F(2, 667) = 0.66$, $p = 0.518$, $\eta_p^2 = 0.002$	$F(2, 667) = 11.29$, $\boldsymbol{p < 0.001}$**,** $\eta_p^2 = 0.017$	$F(2, 667) = 0.06$, $p = 0.946$, $\eta_p^2 = 0.000$
Relationship Skills	$F(2, 667) = 4.31$, $\boldsymbol{p = 0.013}$**,** $\eta_p^2 = 0.013$	$F(2, 667) = 24.18$, $\boldsymbol{p < 0.001}$**,** $\eta_p^2 = 0.035$	$F(2, 667) = 0.17$, $p = 0.843$, $\eta_p^2 = 0.001$
Responsible Decision Making	$F(2, 667) = 3.44$, $\boldsymbol{p = 0.033}$**,** $\eta_p^2 = 0.010$	$F(2, 667) = 14.95$, $\boldsymbol{p < 0.001}$**,** $\eta_p^2 = 0.022$	$F(2, 667) = 0.84$, $p = 0.431$, $\eta_p^2 = 0.003$

Table S15. Teachers' adaptations for online activities using digital resources (Number of Subjects that mention each theme and subtheme).

Adaptations for online activities using digital resources<br>- Themes and subthemes -	Teachers
**Synchronous**	27
Digital Platforms	20
Presentations	14
Virtual Classrooms	6
**Asynchronous**	8
Execution of part of the activity	7
Extra resources	3
